# Synthesis, crystal structure and Hirshfeld analysis of *trans*-bis­{(2*E*)-*N*-phenyl-2-[(2*E*)-3-phenyl-2-propen-1-yl­idene]hydrazinecarbo­thio­amidato-κ^2^ *N* ^1^,*S*}palladium(II)

**DOI:** 10.1107/S2056989023008654

**Published:** 2023-10-05

**Authors:** Ana Paula Lopes de Melo, Bianca Barreto Martins, Leandro Bresolin, Bárbara Tirloni, Adriano Bof de Oliveira

**Affiliations:** a Escola de Química e Alimentos, Universidade Federal do Rio Grande, Av. Itália km 08, Campus Carreiros, 96203-900 Rio Grande-RS, Brazil; b Departamento de Química, Universidade Federal de Santa Maria, Av. Roraima s/n, Campus Universitário, 97105-900 Santa Maria-RS, Brazil; c Departamento de Química, Universidade Federal de Sergipe, Av. Marcelo Deda Chagas s/n, Campus Universitário, 49107-230 São Cristóvão-SE, Brazil; Venezuelan Institute of Scientific Research, Venezuela

**Keywords:** palladium(II) thio­semicarbazone complex, cinnamaldehyde 4-phenyl­thio­semicarbazone, Hirshfeld surface analysis, crystal structure

## Abstract

The reaction of (2*E*)-*N*-phenyl-2-[(2*E*)-3-phenyl-2-propen-1-yl­idene]hydra­zine­carbo­thio­amide (common name: cinnamaldehyde-4-phenyl­thio­semi­carbazone) deprotonated with NaOH in ethanol with an ethano­lic suspension of Pd^II^ chloride in a 2:1 molar ratio yielded the title compound, [Pd(C_16_H_14_N_3_S)_2_]. The anionic ligands act as metal chelators, κ^2^ *N* ^1^ *S*-donors, forming five-membered rings with a *trans*-configuration. The Pd^II^ ion is fourfold coordinated in a slightly distorted square-planar geometry. For each ligand, one H⋯S and one H⋯N intra­molecular inter­actions are observed, with *S*(5) and *S*(6) graph-set motifs. Concerning the H⋯S inter­actions, the coordination sphere resembles a hydrogen-bonded macrocyclic environment-type. In the crystal, the complexes are linked *via* pairs of H⋯S inter­actions, with graph-set motif *R*_2_^2^(8), and building a mono-periodic hydrogen-bonded ribbon along [001]. The Hirshfeld surface analysis indicates that the major contributions for the crystal cohesion are: H⋯H (45.3%), H⋯C/C⋯H (28.0%), H⋯S/S⋯H (8.0%) and H⋯N/N⋯H (7.4%).

## Abstract 2

The synthesis, crystal structure and Hirshfeld surface analysis of a new Pd^II^ cinnamaldehyde 4-thio­semicarbazone homoleptic complex is reported. As a result of H⋯S intra­molecular inter­actions, graph-set motif *S*(5), the coordination sphere resembles a hydrogen-bonded macrocyclic environment-type. In the crystal, the mol­ecules are linked by H⋯S inter­actions, with graph-set motifs 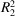 (8), forming a mono-periodic hydrogen-bonded polymer along [001].

## 1. Chemical context

As far as we know, the thio­semicarbazone chemistry can be traced back to the beginning of the 1900s, when a thio­semicarbazide derivative, H_2_N—N(H)C(=S)N*R*_1_ *R*_2_, was used as chemical reagent for the characterization of aldehydes and ketones, *R*_3_ *R*_4_C=O. It was pointed out that the main product of the characterization reaction was a thio­semicarbazone derivative, *R*_3_ *R*_4_C=N—N(H)C(=S)N*R*_1_ *R*_2_ (Freund & Schander, 1902 ▸). In the second half of the 1950s, the use of 4-phenyl­thio­semicarbazide as reagent for the characterization of cinnamaldehyde was reported and the cinnamaldehyde 4-phenyl­thio­semicarbazone mol­ecule, the ligand of the title compound, was the major product of the reaction (Tišler, 1956 ▸).

From early times, as a product of qualitative analysis reactions in the organic chemistry, thio­semicarbazone chemistry emerged as a large class of compounds present in a wide range of scientific disciplines. For example, the cinnamaldehyde 4-phenyl­thio­semicarbazone derivative shows anti-corrosion activity for copper in nitric acid media (Mostafa, 2000 ▸).

One of the most important applications of thio­semicarbazone derivatives is in coordination chemistry. The N—N(H)—C(=S) fragment can be easily deprotonated and the negative charge is then delocalized over the N—N—C—S entity, which enables chemical bonding with many different metal centers, with different Lewis acidity, and a diversity of coordination modes, *e.g.*, chelating and bridging. Complexes with anionic thio­semicarbazone derivatives are more common as a result of the charge density and the geometry adopted by the ligands (Lobana *et al.*, 2009 ▸).

Many complexes with thio­semicarbazone ligands show relevant biological activity. For example, Pd^II^ heteroleptic complexes with a cinnamaldehyde-thio­semicarbazone deriv­ative turned out to be very active on *in vitro* Human Topo­isomerase IIα inhibition, a biological target of prime importance for cancer research (Rocha *et al.*, 2019 ▸). Other Pd^II^ homoleptic and heteroleptic complexes with cinnamaldehyde-thio­semicarbazone as ligands were reported to be active against five human cancer cell lines *in vitro*: colon (Caco-2), cervix (HeLa), hepatocellular (HepG2), breast (MCF-7) and prostate (PC-3) (Nyawadea *et al.*, 2021 ▸). Finally, Ni^II^ homoleptic cinnamaldehyde-4-ethyl­thio­semicarbazone and cinnamaldehyde-4-methyl­thio­semicarbazone derivative complexes showed, also in *in vitro* assays, inhibition of cell growth for two selected human tumour cell lines: breast (MCF-7) and lung (A549) (Farias *et al.*, 2021 ▸).

Another inter­esting approach for cinnamaldehyde-thio­semicarbazone chemistry is the synthesis of nanostructured materials through thermal and solvothermal decomposition techniques, where thio­semicarbazone complexes are employed as single-mol­ecule precursors. It was reported that the thermal and solvothermal decomposition of Zn*L*_2_ and ZnCl_2_(*L*H)_2_ homo- and heteroleptic complexes results in the formation of ZnS nanocrystallites (for this section only, *L* = the anionic form of cinnamaldehyde-thio­semicarbazone and *L*H = the neutral form of it) (Palve & Garje, 2011 ▸). Similarly, Cd^II^ heteroleptic complexes CdCl_2_(*L*H)_2_ and CdI_2_(*L*H)_2_ were used as starting materials to obtain CdS nanoparticles (Pawar *et al.*, 2016 ▸) and CoS or Co_9_S_8_ nanocrystallites were synthesized from Co*L*_2_ and CoCl_2_(*L*H)_2_ homo- and heteroleptic complexes (Pawar & Garje, 2015 ▸).

Motivated by the bioinorganic chemistry and materials science of the cinnamaldehyde-thio­semicarbazone complexes, we report herein the synthesis, crystal structure and Hirshfeld analysis of a new Pd^II^ homoleptic complex where the cinnamaldehyde-4-phenyl­thio­semicarbazone mol­ecules act as anionic ligands. 
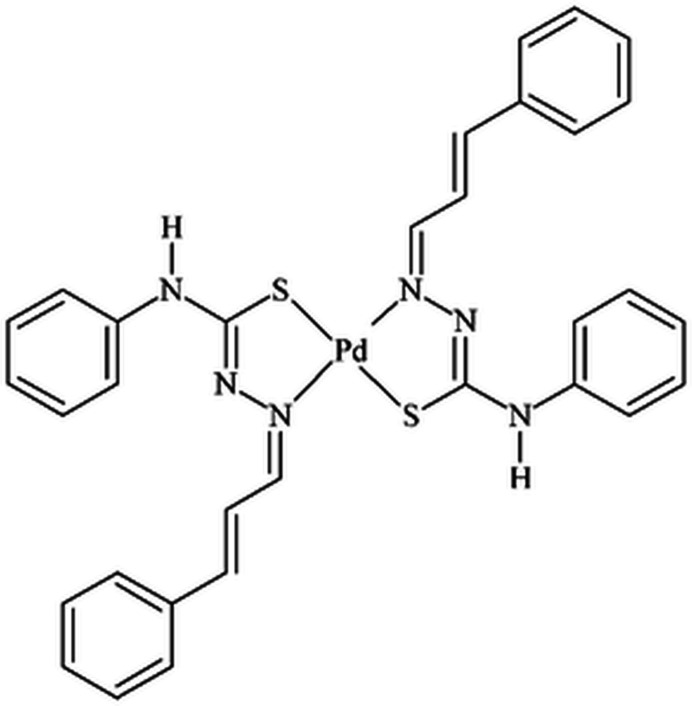


## 2. Structural commentary

**Figure 1:**
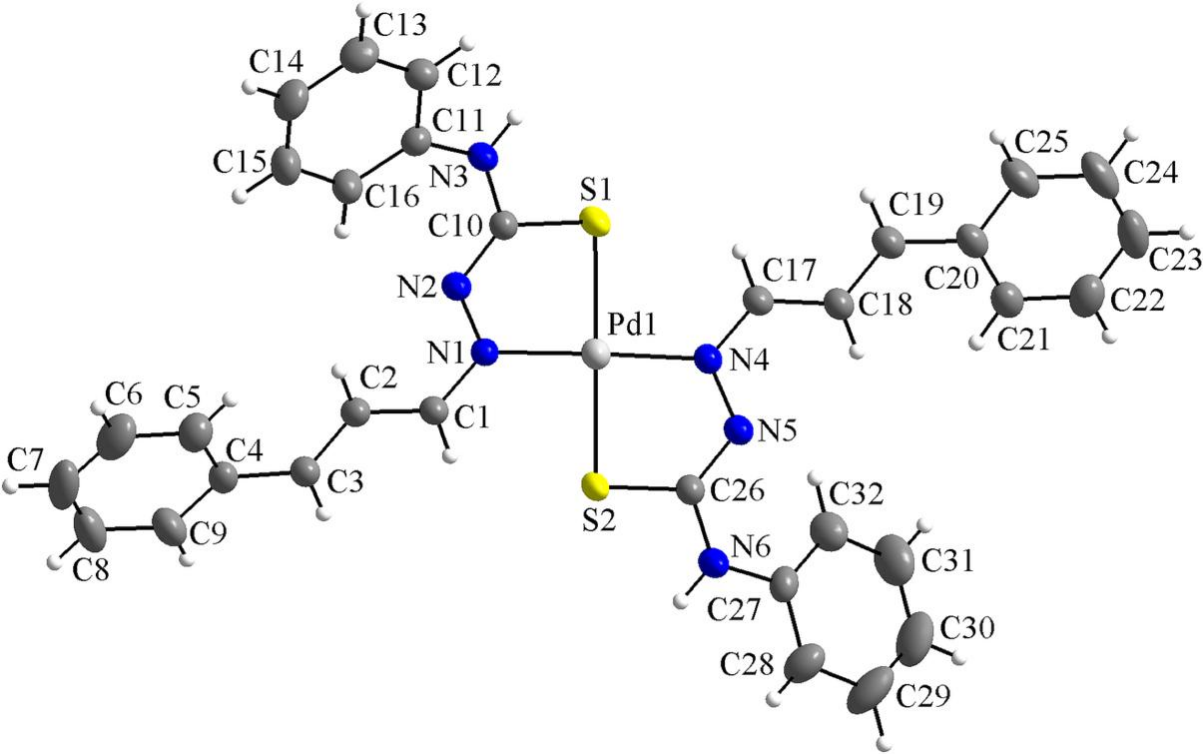
The mol­ecular structure of the title compound, showing the atom labeling and displacement ellipsoids drawn at the 40% probability level.

The asymmetric unit comprises one mol­ecule of the title compound, with all atoms being located in general positions (Fig. 1 ▸). The complex consists of one Pd^II^ metal center and two deprotonated cinnamaldehyde-4-phenyl­thio­semicarbazone ligands, which act as metal chelators, forming five-membered metallarings. The ligands are coordinated through N and S atoms in a *trans*-configuration, κ^2^ *N* ^1^ *S*-donors, and the N1—Pd1—N4 and the S1—Pd1—S2 angles are 178.31 (6) and 177.57 (2)°, respectively. The metal ion is fourfold coordinated in a slightly distorted square-planar geometry. The maximum deviation from the mean plane through the Pd1/N1/N4/S1/S2 fragment is 0.0227 (5) Å for Pd1 and the r.m.s. for the selected atoms is 0.0151 Å. Concerning the geometry of the N—N—C—S entities, the N1—N2—C10—S1 torsion angle is 0.6 (3)°, while N4—N5—C26—S2 is −0.4 (3)°. Both of the ligands are non-planar, with the angle between the mean planes through the C4–C9 and the C11–C16 aromatic rings being 15.7 (1)°, while that between the C20–C25 and the C27–C32 rings is 45.5 (8)°.

**Figure 2:**
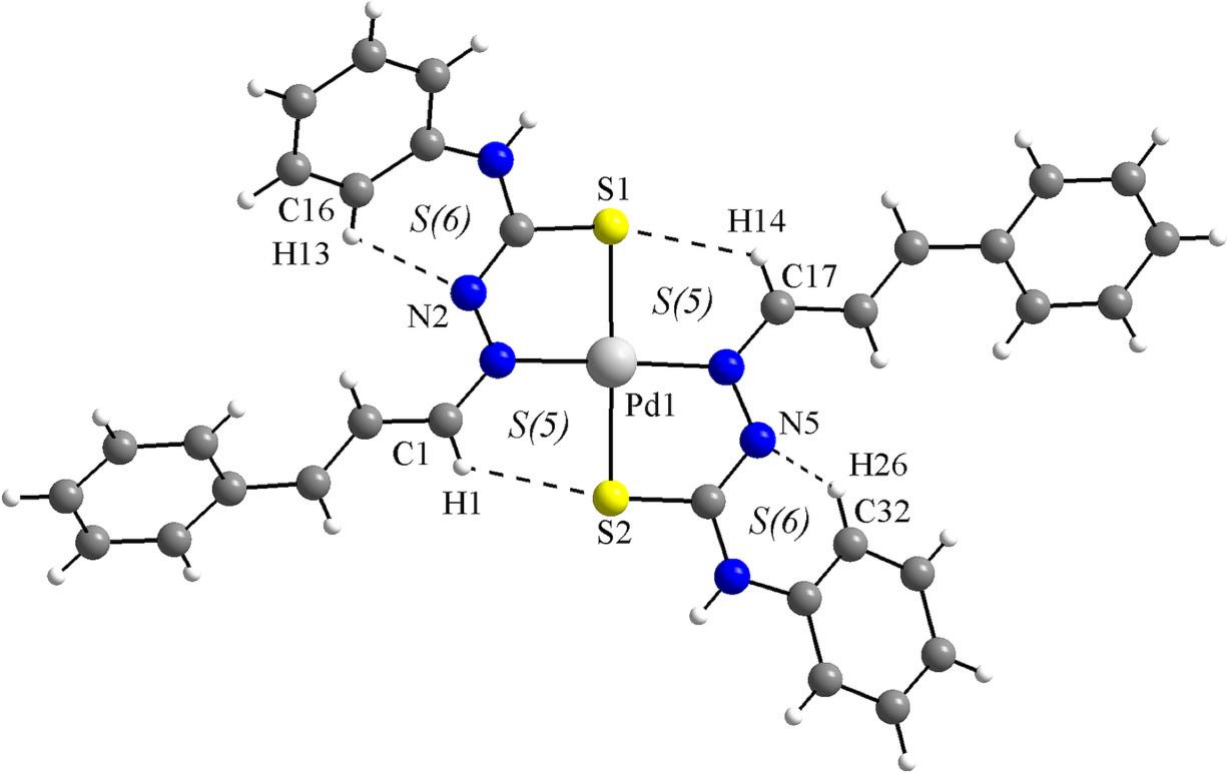
C—H⋯S and C—H⋯N hydrogen intra­molecular inter­actions of the title compound (dashed lines), forming rings of *S*(5) and *S*(6) graph-set motifs. A hydrogen-bonded macrocyclic coordination environment-type can be suggested for the Pd^II^ metal center.

Four intra­molecular hydrogen-bonding inter­actions are observed (Fig. 2 ▸, Table 1 ▸): C1—H1⋯S2 and C17—H14⋯S1, with graph-set motif *S*(5), and C16—H13⋯N2 and C32—H26⋯N5, with graph-set motif *S*(6). Considering the *S*(5) rings, a hydrogen-bonded macrocyclic coordination environment-type can be suggested for the Pd^II^ metal center, while the *S*(6) rings contribute to the stabilization of the mol­ecular structure.

Finally, the anionic form of the ligands was assigned because of the absence of hydrazinic H atoms and the change in the bond lengths of the N—N—C—S entities. For the neutral or free, *i.e.*, non-coordinating thio­semicarbazones, the N—N and C—S bonds have lengths of double-bond character, while the N—C bond shows lengths of single-bond type, which can be written as a N=N(H)—C=S fragment. When the acidic H atom of the hydrazinic fragment is removed, the negative charge is delocalized over the N—N—C—S chain and the bond lengths change to inter­mediate values. Thus, the N—N and the C—S bond lengths assume single-bond character, being longer, and the N—C bond lengths assume double-bond character, being shorter. Information about the bond lengths of the N—N—C—S entities for the cinnamaldehyde-4-phenyl­thio­semicarbazone mol­ecule, C_16_H_15_N_3_S, and the Ni(C_16_H_14_N_3_S)_2_ (Song *et al.*, 2014 ▸) and Pd(C_16_H_14_N_3_S)_2_ complexes, this work, are summarized in Table 2 ▸. These data are in agreement with reported bond lengths values for thio­semicarbazone derivatives (Oliveira *et al.*, 2014 ▸).

## 3. Supra­molecular features

**Figure 3:**
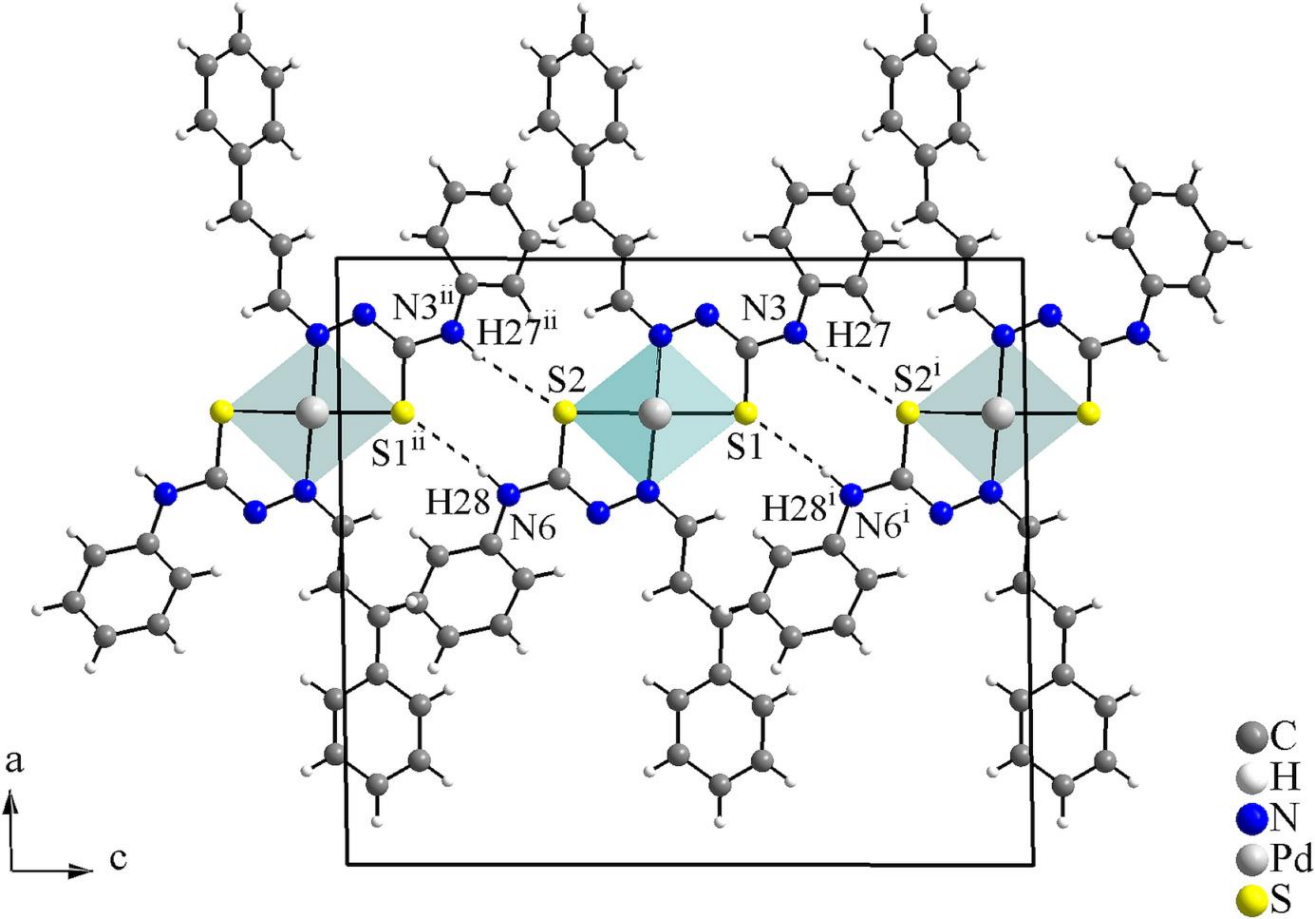
Crystal structure section of the title compound viewed along the *b-*axis. The N—H⋯S inter­actions are drawn as dashed lines, forming rings of 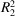 (8) graph-set motif and linking the mol­ecules along the *c-*axis. [Symmetry codes: (i) *x*, −*y* + 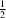 , *z* + 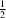 ; (ii) *x*, −*y* + 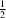 , *z* − 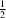 .]

In the crystal, the mol­ecules are connected *via* pairs of N—H⋯S inter­actions with graph-set motif 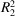 (8), forming a mono-periodic hydrogen-bonded ribbon along [001] (Fig. 3 ▸, Table 1 ▸).

**Figure 4:**
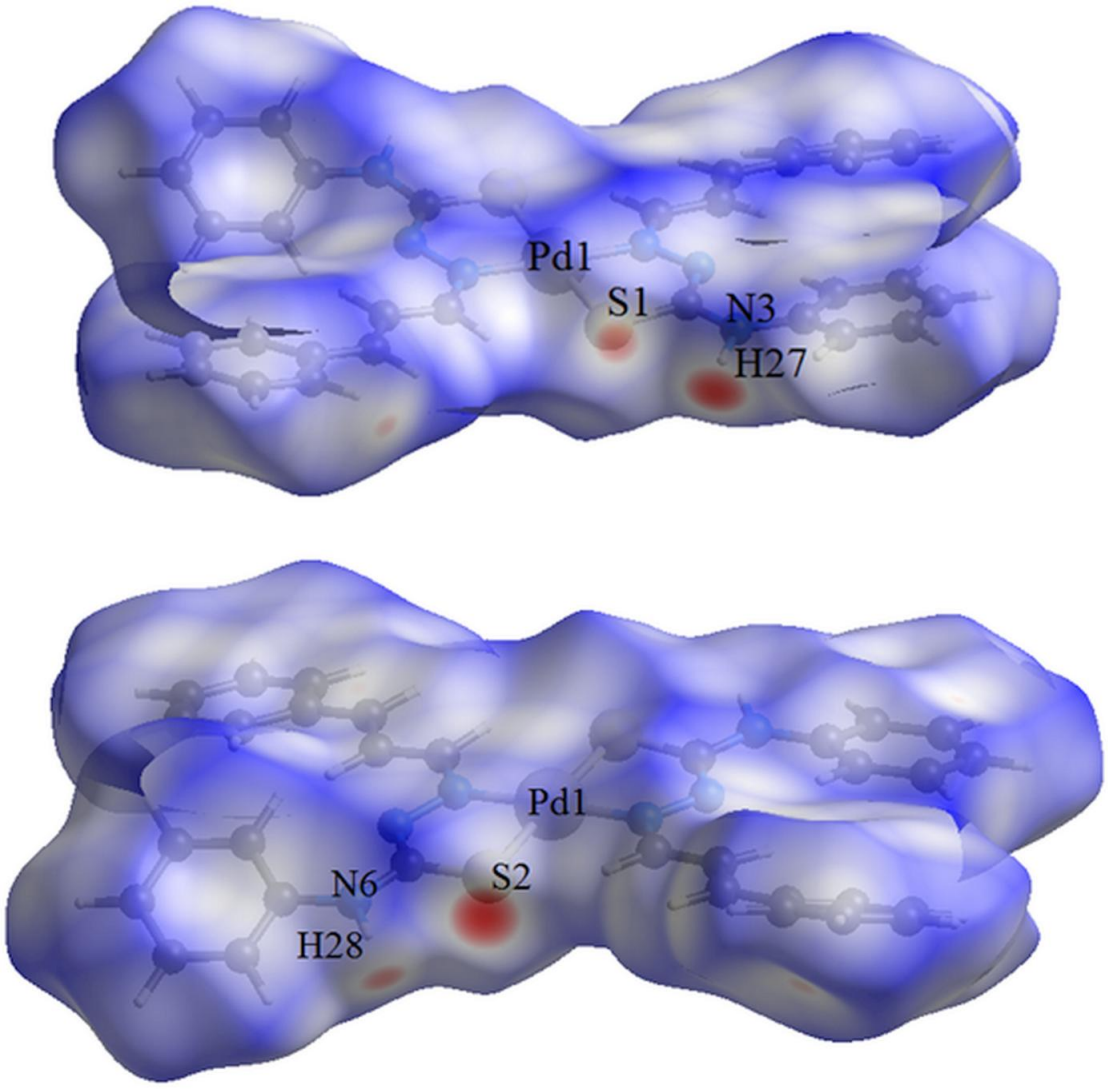
Two side-views in separate figures of the Hirshfeld surface graphical representation (*d*_norm_) for the title compound. The surface is drawn with transparency and simplified for clarity and the regions with strongest inter­molecular inter­actions are shown in magenta. [*d*_norm_ range: −0.289 to 1.415]

**Figure 5:**
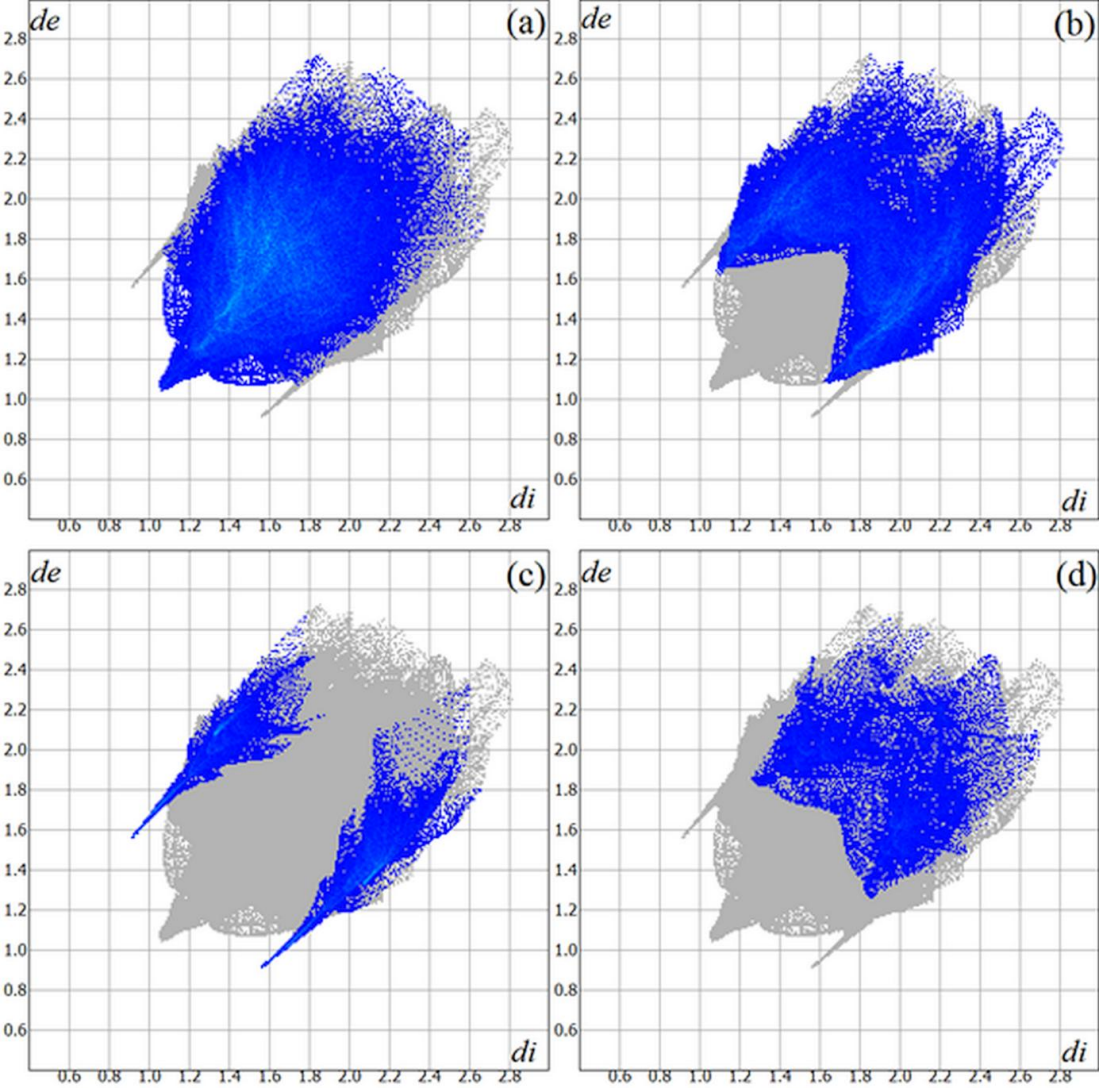
The Hirshfeld surface two-dimensional fingerprint plot for the title compound showing the (*a*) H⋯H, (*b*) H⋯C/C⋯H, (*c*) H⋯S/S⋯H and (*d*) H⋯N/N⋯·H contacts in detail (cyan dots). The contributions of the inter­actions to the crystal cohesion amount to 45.3, 28.0, 8.0 and 7.4%, respectively. The *d*_i_ (*x-*axis) and the *d*_e_ (*y-*axis) values are the closest inter­nal and external distances from given points on the Hirshfeld surface (in Å).

The Hirshfeld surface analysis (Hirshfeld, 1977 ▸) of the crystal structure was performed with *Crystal Explorer* (Wolff *et al.*, 2012 ▸). The graphical representations of the Hirshfeld surface for the title compound are represented using a ball-and-stick model with transparency, in two side-views and separate figures for clarity (Fig. 4 ▸). The locations of the strongest inter­molecular contacts, *i.e*, the regions around the S1, H27, S2 and H28 atoms, are indicated in magenta. These atoms are those involved in the N—H⋯S inter­molecular inter­actions represented in the previous figure (Fig. 3 ▸): N3—H27⋯S2^i^ and N6—H28⋯S1^ii^ [symmetry codes: (i) *x*, −*y* + 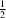 , *z* + 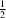 ; (ii) *x*, −*y* + 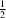 , *z* − 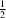 ]. The Hirshfeld surface analysis of the crystal structure also indicates that the most relevant inter­molecular inter­actions for crystal packing are the following: (*a*) H⋯H (45.3%), (*b*) H⋯C/C⋯H (28.0%), (*c*) H⋯S/S⋯H (8.0%) and (*d*) H⋯N/N⋯H (7.4%). The contributions to the crystal packing are shown as two-dimensional Hirshfeld surface fingerprint plots with cyan dots (Fig. 5 ▸). The *d*_i_ (*x-*axis) and the *d*_e_ (*y-*axis) values are the closest inter­nal and external distances from given points on the Hirshfeld surface (in Å).

## 4. Database survey

**Figure 6:**
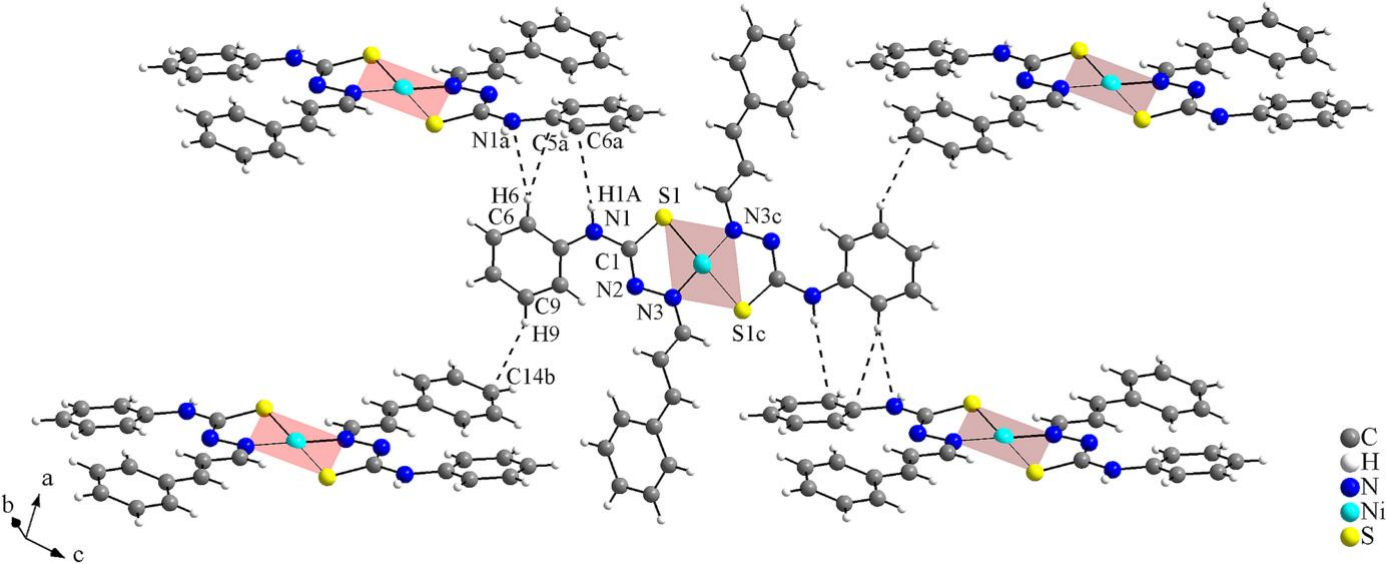
Part of the crystal structure of the reference compound, the centrosymmetric [Ni(C_16_H_14_N_3_S)_2_] complex (Song *et al.*, 2014 ▸). The H⋯C and H⋯N inter­molecular contacts are drawn as dashed lines and the figure is simplified for clarity. [Symmetry codes: (*a*) −*x* + 1, *y* + 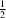 , −*z* + 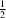 ; (*b*) −*x*, *y* + 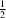 , −*z* + 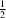 ; (*c*) −*x* + 1, −*y* + 2, −*z* + 1.]

To the best of our knowledge and using database tools such as *SciFinder* ^TM^ (Chemical Abstracts Service, 2023 ▸), there is only one report of the crystal structure of a compound bearing cinnamaldehyde-4-phenyl­thio­semicarbazone as non-coordinated mol­ecule (C_16_H_15_N_3_S) and as a ligand, *viz*. in the homoleptic [Ni(C_16_H_14_N_3_S)_2_] complex (Song *et al.*, 2014 ▸). The asymmetric unit of the reference coordination compound consists of one Ni^II^ ion, which lies on an inversion center, and two deprotonated cinnamaldehyde-4-phenyl­thio­semi­carba­zone ligands, in one of which the atoms are general positions while the second is generated by symmetry (Fig. 6 ▸) [symmetry code: (*c*) −*x* + 1, −*y* + 2, −*z* + 1]. The negative charge of the ligand was assigned by the absence of a hydrazinic H atom and the bond distances in the N—N—C—S chain (please see the remarks in the *Structural commentary* section of this work and also Table 2 ▸). The coordination environment of the Ni^II^ complex is quite similar to that for the Pd^II^ metal center of the title compound: the anionic ligands act as metal chelators, κ^2^ *N* ^1^ *S*-donors, with N and S atoms in *trans*-positions (180°), the metal center is fourfold coordinated in a square-planar geometry and the N—N—C—S entity torsion angle is 1.5 (6)°.

Although the coordination sphere of the Pd^II^ title compound and the Ni^II^ analogue compound are similar, the supra­molecular arrangement of the complexes is totally different. In the crystal, the mol­ecules of the centrosymmetric Ni^II^ coordination compound are linked into a three-dimensional hydrogen-bonded network. The H⋯S inter­molecular inter­actions, like those observed in the Pd^II^ complex (Fig. 3 ▸), are not present in this case and only very weak H⋯C and H⋯N inter­molecular contacts are noted. The values for the hydrogen-bonding of the asymmetric part of the complex amount to: C6—H6⋯C5^*a*^ = 2.90 (5) Å, C6—H6⋯N1^*a*^ = 2.73 (5) Å, C9—H9⋯C14^*b*^ = 2.86 (6) Å and N1—H1*A*⋯C6^*a*^ = 2.90 (7) Å [symmetry codes: (*a*) −*x* + 1, *y* + 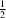 , −*z* + 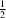 ; (*b*) −*x*, *y* + 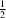 , −*z* + 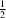 ] (Fig. 6 ▸). The H⋯C and H⋯N distances are slightly above the sum of the van der Waals radii for the respective atoms (Bondi, 1964 ▸; Rowland & Taylor, 1996 ▸) and they are the only inter­molecular contacts observed for the supra­molecular structure of the Ni^II^ complex.

**Figure 7:**
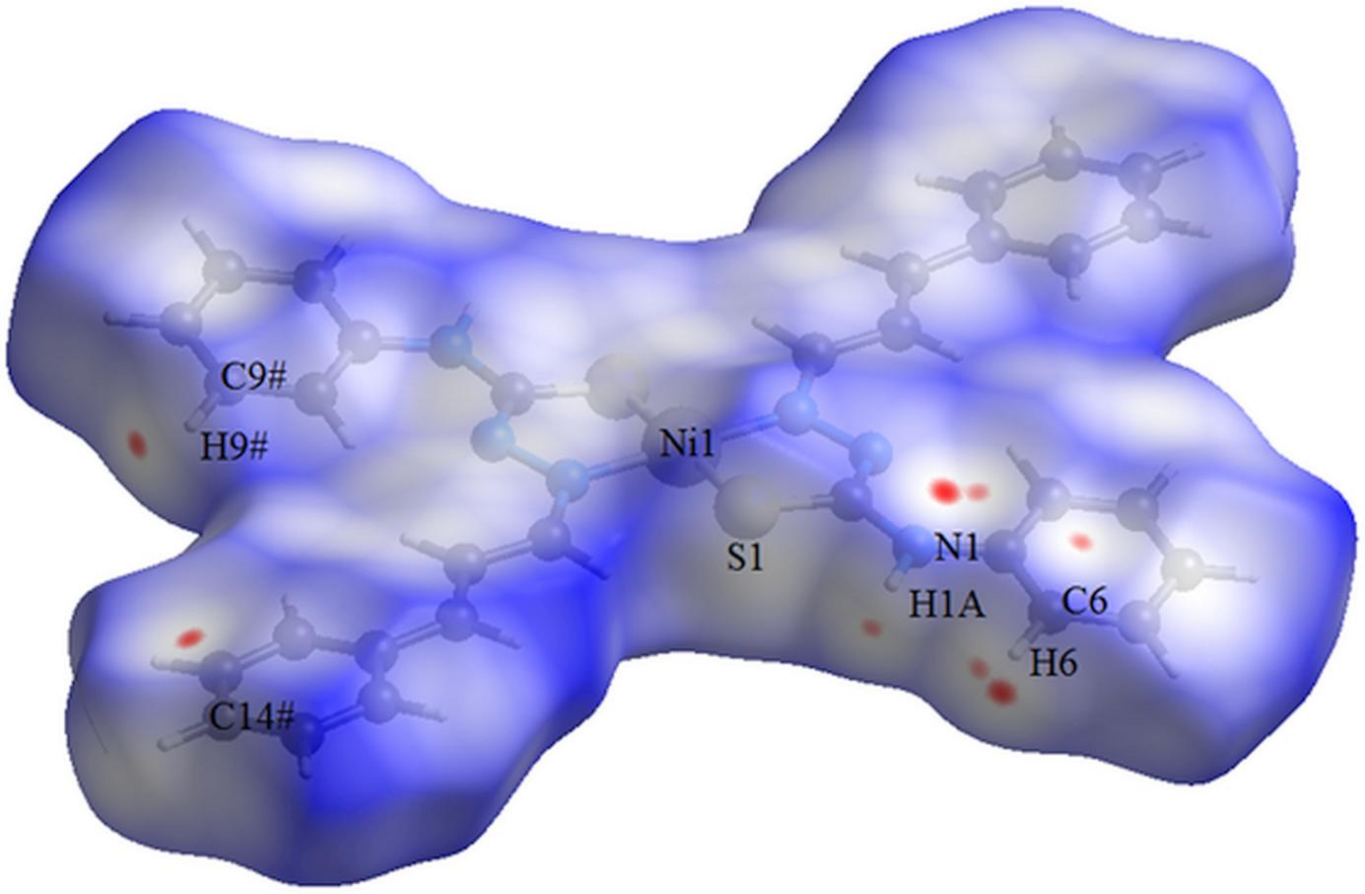
The Hirshfeld surface graphical representation [*d*_norm_ range: −0.045 to 1.492] for the centrosymmetric Ni^II^ complex (Song *et al.*, 2014 ▸). The surface is drawn with transparency and simplified for clarity. The surface regions with strongest inter­molecular contacts are shown in magenta. [Symmetry code: (#) −*x* + 1, -*y+2*, −*z* + 1.]

**Figure 8:**
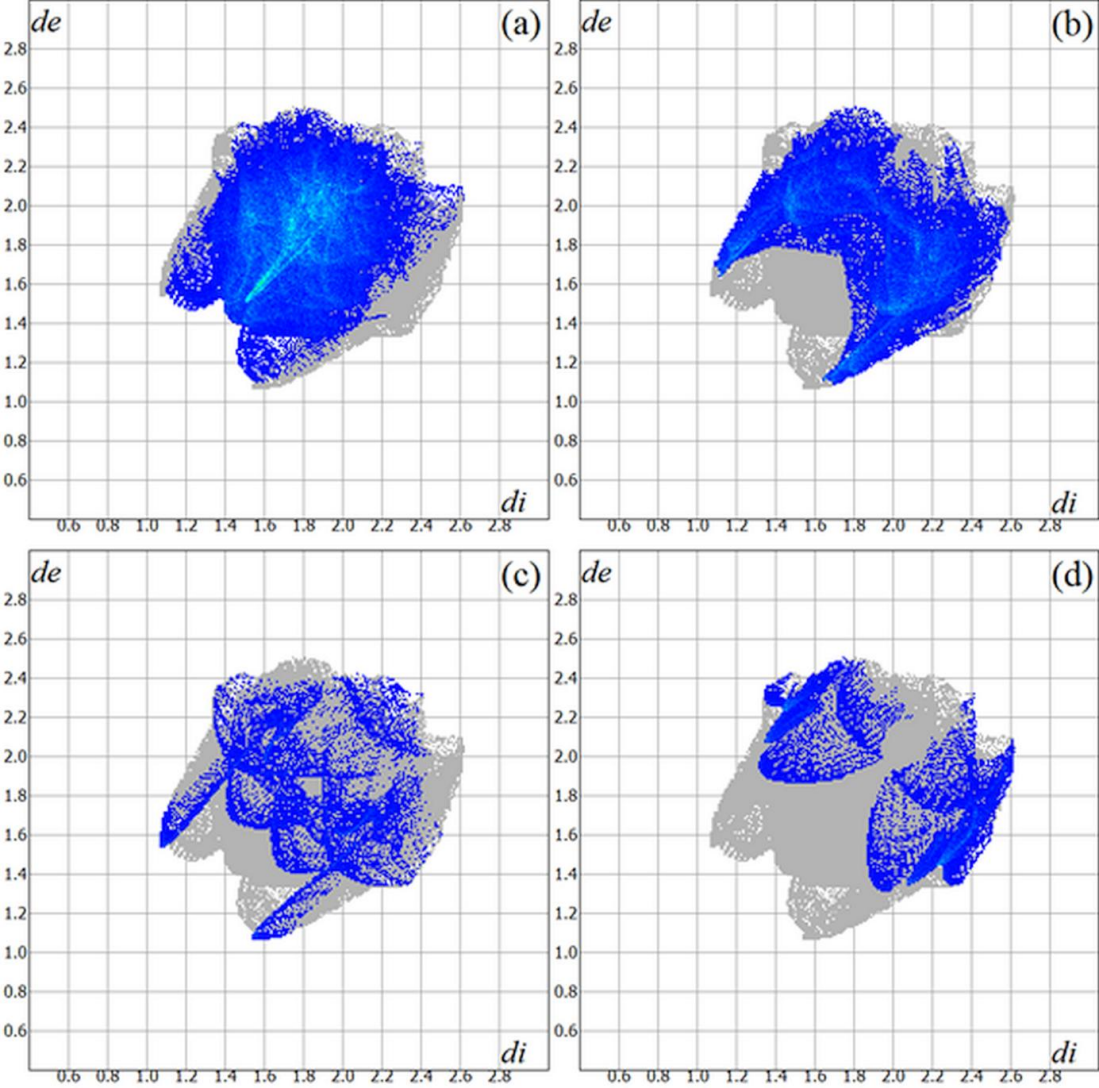
The Hirshfeld surface two-dimensional fingerprint plot for the Ni^II^ coordination compound (Song *et al.*, 2014 ▸) showing the (*a*) H⋯H, (*b*) H⋯C/C⋯H, (*c*) H⋯N/N⋯H and (*d*) H⋯S/S⋯·H contacts in detail (cyan dots). The contributions of the inter­actions to the crystal cohesion amount to 47.4, 27.6, 7.0 and 6.5%, respectively. The *d*_i_ (*x-*axis) and the *d*_e_ (*y-*axis) values are the closest inter­nal and external distances from given points on the Hirshfeld surface (in Å).

The Hirshfeld surface analysis (Hirshfeld, 1977 ▸) of the crystal structure of the Ni^II^ coordination compound was also performed with *CrystalExplorer* (Wolff *et al.*, 2012 ▸). The graphical representation of the Hirshfeld surface is represented using a ball-and-stick model with transparency and the locations of the strongest inter­molecular contacts are draw in magenta, *i.e.*, the regions around the C6, H6, N1, H1*A*, H9^#^ and C14^#^ atoms (Fig. 7 ▸) [symmetry code: (#) −*x* + 1, −*y* + 2, −*z* + 1]. These data are in agreement with the weak H⋯C and H⋯N inter­molecular contacts observed in the previous figure (Fig. 6 ▸). The contributions to the crystal packing are shown as two-dimensional Hirshfeld surface fingerprint plots with cyan dots (Fig. 8 ▸). The Hirshfeld surface analysis of the crystal structure also suggests that the most important inter­molecular inter­actions for crystal packing are the following: (*a*) H⋯H (47.4%), (*b*) H⋯C/C⋯H (27.6%), (*c*) H⋯N/N⋯H (7.0%) and (*d*) H⋯S/S⋯H (6.5%). The *d*_i_ (*x-*axis) and the *d*_e_ (*y-*axis) values are the closest inter­nal and external distances from given points on the Hirshfeld surface contacts (in Å). While for the Pd^II^ title compound and the Ni^II^ reference compound the most important inter­molecular contacts are H⋯H and the H⋯C/C⋯H, the order of importance changes for the H⋯S/S⋯H and H⋯N/N⋯H contacts. For the crystal packing of the Pd^II^ complex, the H⋯S/S⋯H contacts are more important then H⋯N/N⋯H contacts, while for the Ni^II^ complex this order is the opposite.

## 5. Synthesis and crystallization

The starting materials are commercially available and were used without further purification. The synthesis of the ligand was adapted from a previously reported procedure (Freund & Schander, 1902 ▸; Tišler, 1956 ▸). Cinnamaldehyde-4-phenyl­thio­semicarbazone was dissolved in ethanol (4 mmol, 50 mL) and deprotonated with one pellet of NaOH with stirring maintained for 2 h until the solution turned yellow. Simultaneously, an ethano­lic suspension of palladium(II) chloride (2 mmol, 50 mL) was prepared under continuous stirring. A yellow-colored mixture of the ethano­lic solution and the ethano­lic suspension was maintained with stirring at room temperature for 8 h, until the PdCl_2_ was consumed. Orange single crystals suitable for X-ray diffraction were obtained by the slow evaporation of the solvent.

## 6. Refinement

Crystal data, data collection and structure refinement details are summarized in Table 3 ▸. Hydrogen atoms were located in a difference-Fourier map, but were positioned with idealized geometry and refined isotropically using a riding model (HFIX command), with *U*_iso_(H) = 1.2 *U*_eq_(C, N), and with C—H = 0.93 Å and N—H = 0.86 Å.

## Supplementary Material

Crystal structure: contains datablock(s) I, publication_text. DOI: 10.1107/S2056989023008654/zn2032sup1.cif

Structure factors: contains datablock(s) I. DOI: 10.1107/S2056989023008654/zn2032Isup2.hkl

CCDC reference: 2163054

Additional supporting information:  crystallographic information; 3D view; checkCIF report

## Figures and Tables

**Table 1:** Hydrogen-bond geometry (Å, °)

*D*—H⋯*A*	*D*—H	H⋯*A*	*D*⋯*A*	*D*—H⋯*A*
C1—H1⋯S2	0.93	2.60	3.230 (2)	126
C16—H13⋯N2	0.93	2.32	2.887 (3)	119
C17—H14⋯S1	0.93	2.72	3.355 (2)	126
C32—H26⋯N5	0.93	2.39	2.911 (3)	115
N3—H27⋯S2^i^	0.86	2.63	3.4805 (18)	171
N6—H28⋯S1^ii^	0.86	2.84	3.6554 (19)	159

Symmetry codes: (i) 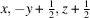 ; (ii) 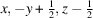 .

**Table 2:** Bond lengths (Å) for the N—N—C—S entities in cinnamaldehyde-4-phenyl­thio­semicarbazone structures: as a neutral mol­ecule and as an anionic ligand

	N—N	N—C	C—S
C_16_H_15_N_3_S^*a*^,^*c*^	1.369 (2)	1.354 (2)	1.6704 (19)
Ni(C_16_H_14_N_3_S)_2_^*b*^,^*c*^	1.405 (5)	1.301 (6)	1.730 (5)
Pd(C_16_H_14_N_3_S)_2_^*b*^,^*d*^	1.390 (2)	1.293 (2)	1.7520 (19)
	1.393 (2)	1.291 (2)	1.7328 (19)

Notes: (*a*) Neutral, non-coordinated form of the cinnamaldehyde 4-phenyl­thio­semicarbazone; (*b*) anionic, coordinated form of the cinnamaldehyde 4-phenyl­thio­semicarbazone; (*c*) Song *et al.* (2014 ▸); (*d*) this work.

**Table 3:** Experimental details

Crystal data	
Chemical formula	[Pd(C_16_H_14_N_3_S)_2_]
*M*_r_	667.12
Crystal system, space group	Monoclinic, *P*2_1_/*c*
Temperature (K)	299
*a*, *b*, *c* (Å)	15.084 (5), 11.418 (4), 17.097 (6)
β (°)	91.097 (9)
*V* (Å^3^)	2944.0 (16)
*Z*	4
Radiation type	Mo *K*α
μ (mm^−1^)	0.81
Crystal size (mm)	0.25 × 0.18 × 0.11

Data collection	
Diffractometer	Bruker D8 Venture Photon 100 area detector diffractometer
Absorption correction	Multi-scan (*SADABS*; Krause *et al.*, 2015 ▸)
*T*_min_, *T*_max_	0.699, 0.746
No. of measured, independent and observed [*I* > 2σ(*I*)] reflections	87933, 7344, 6204
*R*_int_	0.042
(sin θ/λ)_max_ (Å^−1^)	0.668

Refinement	
*R*[*F* ^2^ > 2σ(*F* ^2^)], *wR*(*F* ^2^), *S*	0.026, 0.063, 1.05
No. of reflections	7344
No. of parameters	370
H-atom treatment	H-atom parameters constrained
Δρ_max_, Δρ_min_ (e Å^−3^)	0.34, −0.50

Computer programs: *APEX3* and *SAINT* (Bruker, 2015 ▸), *SHELXT2014/5* (Sheldrick, 2015*a* ▸), *SHELXL2018/3* (Sheldrick, 2015*b* ▸), *DIAMOND* (Brandenburg, 2006 ▸), *CrystalExplorer* (Wolff *et al.*, 2012 ▸), *WinGX* (Farrugia, 2012 ▸), *publCIF* (Westrip, 2010 ▸) and *enCIFer* (Allen *et al.*, 2004 ▸).

## supplementary crystallographic information

### Crystal data

**Table:**

[Pd(C_16_H_14_N_3_S)_2_]	*F*(000) = 1360
*M**_r_* = 667.12	*D*_x_ = 1.505 Mg m^−^^3^
Monoclinic, *P*2_1_/*c*	Mo *K*α radiation, λ = 0.71073 Å
*a* = 15.084 (5) Å	Cell parameters from 9138 reflections
*b* = 11.418 (4) Å	θ = 2.2–28.3°
*c* = 17.097 (6) Å	µ = 0.81 mm^−^^1^
β = 91.097 (9)°	*T* = 299 K
*V* = 2944.0 (16) Å^3^	Block, orange
*Z* = 4	0.25 × 0.18 × 0.11 mm

### Data collection

**Table:**

Bruker D8 Venture Photon 100 area detector diffractometer	6204 reflections with *I* > 2σ(*I*)
Radiation source: microfocus X ray tube	*R*_int_ = 0.042
φ and ω scans	θ_max_ = 28.4°, θ_min_ = 2.2°
Absorption correction: multi-scan (SADABS; Krause *et al.*, 2015)	*h* = −20→20
*T*_min_ = 0.699, *T*_max_ = 0.746	*k* = −15→15
87933 measured reflections	*l* = −22→22
7344 independent reflections	

### Refinement

**Table:**

Refinement on *F*^2^	Primary atom site location: structure-invariant direct methods
Least-squares matrix: full	Hydrogen site location: inferred from neighbouring sites
*R*[*F*^2^ > 2σ(*F*^2^)] = 0.026	H-atom parameters constrained
*wR*(*F*^2^) = 0.063	*w* = 1/[σ^2^(*F*_o_^2^) + (0.0241*P*)^2^ + 1.5227*P*] where *P* = (*F*_o_^2^ + 2*F*_c_^2^)/3
*S* = 1.05	(Δ/σ)_max_ = 0.003
7344 reflections	Δρ_max_ = 0.34 e Å^−^^3^
370 parameters	Δρ_min_ = −0.50 e Å^−^^3^
0 restraints	

### Special details

**Table:**

Geometry. All esds (except the esd in the dihedral angle between two l.s. planes) are estimated using the full covariance matrix. The cell esds are taken into account individually in the estimation of esds in distances, angles and torsion angles; correlations between esds in cell parameters are only used when they are defined by crystal symmetry. An approximate (isotropic) treatment of cell esds is used for estimating esds involving l.s. planes.

### Fractional atomic coordinates and isotropic or equivalent isotropic displacement parameters (Å^2^)

**Table:**

	*x*	*y*	*z*	*U*_iso_*/*U*_eq_	
C1	0.93114 (12)	0.25646 (18)	0.41478 (10)	0.0408 (4)	
H1	0.911357	0.222755	0.368099	0.049*	
C2	1.02188 (12)	0.29262 (18)	0.41878 (11)	0.0429 (4)	
H2	1.042322	0.334403	0.462190	0.052*	
C3	1.07777 (12)	0.26780 (19)	0.36174 (11)	0.0434 (4)	
H3	1.054715	0.228656	0.318196	0.052*	
C4	1.17186 (12)	0.29659 (18)	0.36156 (11)	0.0436 (4)	
C5	1.20873 (15)	0.3801 (2)	0.41107 (14)	0.0566 (5)	
H4	1.172634	0.422924	0.444057	0.068*	
C6	1.29897 (17)	0.4000 (3)	0.41148 (17)	0.0738 (8)	
H5	1.323135	0.457206	0.444183	0.089*	
C7	1.35360 (16)	0.3362 (3)	0.36419 (19)	0.0765 (8)	
H6	1.414532	0.348617	0.366163	0.092*	
C8	1.31865 (17)	0.2555 (3)	0.31492 (18)	0.0745 (8)	
H7	1.355493	0.212955	0.282440	0.089*	
C9	1.22814 (15)	0.2358 (2)	0.31256 (14)	0.0613 (6)	
H8	1.204591	0.181127	0.277638	0.074*	
C10	0.85343 (12)	0.31504 (17)	0.59615 (10)	0.0377 (4)	
C11	0.95766 (12)	0.42049 (17)	0.68829 (10)	0.0397 (4)	
C12	0.95571 (14)	0.48633 (19)	0.75646 (11)	0.0466 (5)	
H9	0.904012	0.489377	0.785175	0.056*	
C13	1.02970 (15)	0.5473 (2)	0.78205 (13)	0.0559 (5)	
H10	1.027308	0.591307	0.827731	0.067*	
C14	1.10674 (15)	0.5437 (2)	0.74084 (14)	0.0584 (6)	
H11	1.156512	0.585126	0.757969	0.070*	
C15	1.10894 (14)	0.4777 (2)	0.67371 (14)	0.0597 (6)	
H12	1.161063	0.474407	0.645610	0.072*	
C16	1.03566 (13)	0.4160 (2)	0.64695 (12)	0.0516 (5)	
H13	1.038671	0.371677	0.601430	0.062*	
C17	0.55839 (12)	0.16720 (18)	0.50233 (11)	0.0416 (4)	
H14	0.578917	0.185524	0.552491	0.050*	
C18	0.46597 (12)	0.14045 (18)	0.49358 (11)	0.0429 (4)	
H15	0.444566	0.115183	0.445071	0.052*	
C19	0.40947 (13)	0.15030 (19)	0.55209 (12)	0.0472 (5)	
H16	0.433316	0.172755	0.600361	0.057*	
C20	0.31419 (13)	0.12958 (18)	0.54837 (12)	0.0454 (4)	
C21	0.27211 (14)	0.0746 (2)	0.48604 (13)	0.0513 (5)	
H17	0.305174	0.047797	0.444312	0.062*	
C22	0.18134 (15)	0.0590 (2)	0.48519 (16)	0.0644 (6)	
H18	0.153934	0.021536	0.442935	0.077*	
C23	0.13142 (16)	0.0979 (3)	0.54540 (18)	0.0713 (8)	
H19	0.070293	0.087173	0.544212	0.086*	
C24	0.17159 (17)	0.1526 (3)	0.60741 (18)	0.0739 (8)	
H20	0.137628	0.180199	0.648339	0.089*	
C25	0.26240 (16)	0.1673 (2)	0.60978 (15)	0.0653 (6)	
H21	0.289272	0.202928	0.653032	0.078*	
C26	0.63696 (12)	0.14116 (18)	0.31857 (11)	0.0406 (4)	
C27	0.52836 (12)	0.06730 (19)	0.21755 (12)	0.0456 (5)	
C28	0.50883 (17)	0.0809 (2)	0.13948 (14)	0.0657 (7)	
H22	0.547569	0.121832	0.107827	0.079*	
C29	0.4319 (2)	0.0341 (3)	0.10764 (19)	0.0878 (10)	
H23	0.418657	0.044846	0.054772	0.105*	
C30	0.37560 (18)	−0.0272 (3)	0.1525 (2)	0.0837 (9)	
H24	0.323855	−0.058496	0.130698	0.100*	
C31	0.39511 (18)	−0.0431 (3)	0.23033 (19)	0.0803 (8)	
H25	0.356585	−0.085945	0.261047	0.096*	
C32	0.47140 (16)	0.0038 (2)	0.26388 (15)	0.0633 (6)	
H26	0.484228	−0.007087	0.316803	0.076*	
N1	0.87363 (10)	0.26614 (14)	0.47008 (8)	0.0375 (3)	
N2	0.90767 (10)	0.31587 (16)	0.53850 (9)	0.0428 (4)	
N3	0.87873 (10)	0.36111 (16)	0.66658 (9)	0.0442 (4)	
H27	0.841082	0.352842	0.703313	0.053*	
N4	0.61600 (10)	0.16837 (14)	0.44748 (9)	0.0383 (3)	
N5	0.58090 (10)	0.13619 (16)	0.37463 (9)	0.0437 (4)	
N6	0.61038 (11)	0.11296 (18)	0.24427 (9)	0.0521 (5)	
H28	0.649000	0.124627	0.208750	0.063*	
Pd1	0.74528 (2)	0.21629 (2)	0.46039 (2)	0.03379 (5)	
S1	0.74605 (3)	0.25655 (5)	0.59231 (3)	0.04797 (12)	
S2	0.74676 (3)	0.18416 (6)	0.32859 (3)	0.04861 (13)	

### Atomic displacement parameters (Å^2^)

**Table:**

	*U*^11^	*U*^22^	*U*^33^	*U*^12^	*U*^13^	*U*^23^
C1	0.0320 (9)	0.0589 (11)	0.0314 (9)	−0.0018 (8)	0.0020 (7)	−0.0014 (8)
C2	0.0327 (9)	0.0625 (12)	0.0337 (9)	−0.0045 (8)	−0.0002 (7)	0.0005 (8)
C3	0.0337 (9)	0.0606 (12)	0.0358 (9)	−0.0037 (9)	0.0015 (7)	0.0006 (9)
C4	0.0326 (9)	0.0575 (12)	0.0407 (10)	−0.0026 (8)	0.0018 (7)	0.0101 (9)
C5	0.0445 (12)	0.0656 (14)	0.0596 (13)	−0.0060 (10)	−0.0040 (10)	0.0032 (11)
C6	0.0551 (15)	0.0816 (18)	0.0838 (18)	−0.0223 (14)	−0.0209 (14)	0.0151 (15)
C7	0.0348 (12)	0.094 (2)	0.100 (2)	−0.0080 (13)	−0.0040 (13)	0.0419 (18)
C8	0.0416 (13)	0.097 (2)	0.0858 (19)	0.0078 (13)	0.0206 (13)	0.0207 (17)
C9	0.0445 (12)	0.0805 (17)	0.0594 (14)	−0.0030 (11)	0.0151 (10)	−0.0011 (12)
C10	0.0314 (9)	0.0475 (10)	0.0343 (9)	0.0002 (7)	0.0017 (7)	−0.0005 (7)
C11	0.0351 (9)	0.0486 (10)	0.0354 (9)	0.0000 (8)	−0.0029 (7)	0.0001 (8)
C12	0.0446 (11)	0.0557 (12)	0.0396 (10)	−0.0024 (9)	0.0016 (8)	−0.0032 (9)
C13	0.0578 (13)	0.0596 (13)	0.0499 (12)	−0.0054 (11)	−0.0067 (10)	−0.0103 (10)
C14	0.0443 (12)	0.0655 (14)	0.0650 (14)	−0.0103 (10)	−0.0128 (10)	−0.0005 (11)
C15	0.0341 (10)	0.0826 (17)	0.0624 (14)	−0.0034 (11)	0.0010 (10)	−0.0032 (12)
C16	0.0361 (10)	0.0735 (14)	0.0453 (11)	0.0009 (10)	0.0016 (8)	−0.0096 (10)
C17	0.0360 (9)	0.0542 (11)	0.0347 (9)	−0.0016 (8)	0.0036 (7)	−0.0037 (8)
C18	0.0356 (9)	0.0542 (11)	0.0392 (10)	−0.0007 (8)	0.0053 (8)	−0.0008 (8)
C19	0.0413 (10)	0.0586 (12)	0.0420 (10)	−0.0018 (9)	0.0078 (8)	−0.0009 (9)
C20	0.0390 (10)	0.0505 (11)	0.0471 (11)	0.0018 (8)	0.0116 (8)	0.0071 (9)
C21	0.0415 (11)	0.0609 (13)	0.0516 (12)	0.0024 (10)	0.0072 (9)	0.0081 (10)
C22	0.0468 (13)	0.0740 (16)	0.0722 (16)	−0.0050 (12)	−0.0071 (11)	0.0167 (13)
C23	0.0379 (12)	0.0804 (18)	0.096 (2)	0.0040 (12)	0.0149 (13)	0.0283 (16)
C24	0.0539 (14)	0.0804 (18)	0.089 (2)	0.0055 (13)	0.0362 (14)	0.0086 (16)
C25	0.0539 (14)	0.0782 (16)	0.0645 (15)	−0.0027 (12)	0.0246 (11)	−0.0050 (13)
C26	0.0315 (9)	0.0554 (11)	0.0351 (9)	−0.0013 (8)	0.0021 (7)	−0.0016 (8)
C27	0.0323 (9)	0.0553 (12)	0.0488 (11)	0.0009 (8)	−0.0040 (8)	−0.0101 (9)
C28	0.0631 (15)	0.0751 (16)	0.0582 (14)	−0.0127 (13)	−0.0205 (12)	0.0074 (12)
C29	0.084 (2)	0.095 (2)	0.083 (2)	−0.0132 (18)	−0.0477 (17)	0.0046 (17)
C30	0.0520 (15)	0.089 (2)	0.109 (2)	−0.0128 (14)	−0.0269 (16)	−0.0201 (18)
C31	0.0566 (15)	0.087 (2)	0.098 (2)	−0.0283 (14)	0.0074 (15)	−0.0243 (17)
C32	0.0520 (13)	0.0787 (16)	0.0592 (14)	−0.0154 (12)	0.0031 (11)	−0.0121 (12)
N1	0.0299 (7)	0.0523 (9)	0.0303 (7)	−0.0014 (6)	0.0009 (6)	−0.0023 (6)
N2	0.0324 (8)	0.0645 (10)	0.0316 (8)	−0.0058 (7)	0.0017 (6)	−0.0059 (7)
N3	0.0348 (8)	0.0659 (11)	0.0319 (8)	−0.0070 (8)	0.0045 (6)	−0.0067 (7)
N4	0.0304 (7)	0.0496 (9)	0.0349 (8)	−0.0023 (7)	0.0030 (6)	−0.0023 (7)
N5	0.0322 (8)	0.0646 (11)	0.0342 (8)	−0.0057 (7)	0.0027 (6)	−0.0055 (7)
N6	0.0341 (8)	0.0878 (14)	0.0346 (8)	−0.0110 (9)	0.0028 (7)	−0.0081 (8)
Pd1	0.02574 (7)	0.04563 (8)	0.03007 (7)	−0.00040 (5)	0.00212 (5)	−0.00124 (5)
S1	0.0344 (2)	0.0749 (3)	0.0349 (2)	−0.0119 (2)	0.00699 (18)	−0.0100 (2)
S2	0.0275 (2)	0.0866 (4)	0.0319 (2)	−0.0068 (2)	0.00370 (17)	−0.0071 (2)

### Geometric parameters (Å, º)

**Table:**

C1—N1	1.300 (2)	C18—H15	0.9300
C1—C2	1.430 (3)	C19—C20	1.457 (3)
C1—H1	0.9300	C19—H16	0.9300
C2—C3	1.332 (3)	C20—C21	1.381 (3)
C2—H2	0.9300	C20—C25	1.389 (3)
C3—C4	1.457 (3)	C21—C22	1.380 (3)
C3—H3	0.9300	C21—H17	0.9300
C4—C5	1.385 (3)	C22—C23	1.361 (4)
C4—C9	1.389 (3)	C22—H18	0.9300
C5—C6	1.380 (3)	C23—C24	1.363 (4)
C5—H4	0.9300	C23—H19	0.9300
C6—C7	1.374 (4)	C24—C25	1.380 (3)
C6—H5	0.9300	C24—H20	0.9300
C7—C8	1.349 (4)	C25—H21	0.9300
C7—H6	0.9300	C26—N5	1.291 (2)
C8—C9	1.383 (3)	C26—N6	1.363 (2)
C8—H7	0.9300	C26—S2	1.7328 (19)
C9—H8	0.9300	C27—C28	1.370 (3)
C10—N2	1.293 (2)	C27—C32	1.384 (3)
C10—N3	1.362 (2)	C27—N6	1.410 (2)
C10—S1	1.7520 (19)	C28—C29	1.380 (3)
C11—C16	1.385 (3)	C28—H22	0.9300
C11—C12	1.388 (3)	C29—C30	1.351 (4)
C11—N3	1.413 (2)	C29—H23	0.9300
C12—C13	1.380 (3)	C30—C31	1.369 (4)
C12—H9	0.9300	C30—H24	0.9300
C13—C14	1.371 (3)	C31—C32	1.384 (3)
C13—H10	0.9300	C31—H25	0.9300
C14—C15	1.374 (3)	C32—H26	0.9300
C14—H11	0.9300	N1—N2	1.390 (2)
C15—C16	1.381 (3)	N1—Pd1	2.0217 (16)
C15—H12	0.9300	N3—H27	0.8600
C16—H13	0.9300	N4—N5	1.393 (2)
C17—N4	1.291 (2)	N4—Pd1	2.0333 (16)
C17—C18	1.432 (3)	N6—H28	0.8600
C17—H14	0.9300	Pd1—S2	2.2836 (9)
C18—C19	1.331 (3)	Pd1—S1	2.3016 (9)
			
N1—C1—C2	126.30 (17)	C25—C20—C19	119.0 (2)
N1—C1—H1	116.8	C22—C21—C20	120.5 (2)
C2—C1—H1	116.8	C22—C21—H17	119.7
C3—C2—C1	121.49 (18)	C20—C21—H17	119.7
C3—C2—H2	119.3	C23—C22—C21	120.9 (3)
C1—C2—H2	119.3	C23—C22—H18	119.6
C2—C3—C4	125.73 (19)	C21—C22—H18	119.6
C2—C3—H3	117.1	C22—C23—C24	119.6 (2)
C4—C3—H3	117.1	C22—C23—H19	120.2
C5—C4—C9	118.0 (2)	C24—C23—H19	120.2
C5—C4—C3	122.27 (19)	C23—C24—C25	120.3 (2)
C9—C4—C3	119.7 (2)	C23—C24—H20	119.8
C6—C5—C4	120.0 (2)	C25—C24—H20	119.8
C6—C5—H4	120.0	C24—C25—C20	120.8 (3)
C4—C5—H4	120.0	C24—C25—H21	119.6
C7—C6—C5	120.8 (3)	C20—C25—H21	119.6
C7—C6—H5	119.6	N5—C26—N6	119.74 (17)
C5—C6—H5	119.6	N5—C26—S2	125.25 (14)
C8—C7—C6	119.9 (2)	N6—C26—S2	115.00 (13)
C8—C7—H6	120.1	C28—C27—C32	119.5 (2)
C6—C7—H6	120.1	C28—C27—N6	116.4 (2)
C7—C8—C9	120.2 (3)	C32—C27—N6	123.9 (2)
C7—C8—H7	119.9	C27—C28—C29	120.3 (3)
C9—C8—H7	119.9	C27—C28—H22	119.9
C8—C9—C4	121.0 (3)	C29—C28—H22	119.9
C8—C9—H8	119.5	C30—C29—C28	120.6 (3)
C4—C9—H8	119.5	C30—C29—H23	119.7
N2—C10—N3	120.03 (17)	C28—C29—H23	119.7
N2—C10—S1	124.91 (14)	C29—C30—C31	119.7 (2)
N3—C10—S1	115.06 (13)	C29—C30—H24	120.2
C16—C11—C12	118.73 (18)	C31—C30—H24	120.2
C16—C11—N3	124.58 (18)	C30—C31—C32	120.9 (3)
C12—C11—N3	116.68 (17)	C30—C31—H25	119.6
C13—C12—C11	120.6 (2)	C32—C31—H25	119.6
C13—C12—H9	119.7	C31—C32—C27	119.0 (2)
C11—C12—H9	119.7	C31—C32—H26	120.5
C14—C13—C12	120.7 (2)	C27—C32—H26	120.5
C14—C13—H10	119.7	C1—N1—N2	113.95 (15)
C12—C13—H10	119.7	C1—N1—Pd1	124.60 (13)
C13—C14—C15	118.7 (2)	N2—N1—Pd1	121.45 (11)
C13—C14—H11	120.7	C10—N2—N1	114.18 (15)
C15—C14—H11	120.7	C10—N3—C11	129.63 (16)
C14—C15—C16	121.6 (2)	C10—N3—H27	115.2
C14—C15—H12	119.2	C11—N3—H27	115.2
C16—C15—H12	119.2	C17—N4—N5	113.40 (15)
C15—C16—C11	119.6 (2)	C17—N4—Pd1	125.56 (13)
C15—C16—H13	120.2	N5—N4—Pd1	121.01 (11)
C11—C16—H13	120.2	C26—N5—N4	114.13 (15)
N4—C17—C18	126.43 (17)	C26—N6—C27	129.03 (17)
N4—C17—H14	116.8	C26—N6—H28	115.5
C18—C17—H14	116.8	C27—N6—H28	115.5
C19—C18—C17	122.63 (19)	N1—Pd1—N4	178.31 (6)
C19—C18—H15	118.7	N1—Pd1—S2	95.66 (4)
C17—C18—H15	118.7	N4—Pd1—S2	82.94 (4)
C18—C19—C20	126.8 (2)	N1—Pd1—S1	82.92 (4)
C18—C19—H16	116.6	N4—Pd1—S1	98.45 (4)
C20—C19—H16	116.6	S2—Pd1—S1	177.57 (2)
C21—C20—C25	117.9 (2)	C10—S1—Pd1	95.74 (6)
C21—C20—C19	123.10 (18)	C26—S2—Pd1	96.65 (6)
			
N1—C1—C2—C3	172.9 (2)	C32—C27—C28—C29	1.5 (4)
C1—C2—C3—C4	−177.50 (19)	N6—C27—C28—C29	177.0 (3)
C2—C3—C4—C5	−17.8 (3)	C27—C28—C29—C30	−1.1 (5)
C2—C3—C4—C9	159.6 (2)	C28—C29—C30—C31	0.0 (5)
C9—C4—C5—C6	−0.8 (3)	C29—C30—C31—C32	0.6 (5)
C3—C4—C5—C6	176.7 (2)	C30—C31—C32—C27	−0.1 (4)
C4—C5—C6—C7	−1.2 (4)	C28—C27—C32—C31	−0.9 (4)
C5—C6—C7—C8	2.0 (4)	N6—C27—C32—C31	−176.0 (2)
C6—C7—C8—C9	−0.8 (4)	C2—C1—N1—N2	−0.7 (3)
C7—C8—C9—C4	−1.3 (4)	C2—C1—N1—Pd1	178.74 (16)
C5—C4—C9—C8	2.0 (4)	N3—C10—N2—N1	179.95 (17)
C3—C4—C9—C8	−175.5 (2)	S1—C10—N2—N1	0.6 (3)
C16—C11—C12—C13	0.8 (3)	C1—N1—N2—C10	−173.45 (18)
N3—C11—C12—C13	−179.92 (19)	Pd1—N1—N2—C10	7.1 (2)
C11—C12—C13—C14	−0.3 (3)	N2—C10—N3—C11	4.6 (3)
C12—C13—C14—C15	−0.3 (4)	S1—C10—N3—C11	−175.92 (16)
C13—C14—C15—C16	0.4 (4)	C16—C11—N3—C10	−18.5 (3)
C14—C15—C16—C11	0.2 (4)	C12—C11—N3—C10	162.2 (2)
C12—C11—C16—C15	−0.7 (3)	C18—C17—N4—N5	−2.1 (3)
N3—C11—C16—C15	−180.0 (2)	C18—C17—N4—Pd1	176.05 (16)
N4—C17—C18—C19	−174.5 (2)	N6—C26—N5—N4	−179.31 (18)
C17—C18—C19—C20	177.5 (2)	S2—C26—N5—N4	−0.4 (3)
C18—C19—C20—C21	13.0 (4)	C17—N4—N5—C26	177.55 (18)
C18—C19—C20—C25	−166.5 (2)	Pd1—N4—N5—C26	−0.7 (2)
C25—C20—C21—C22	0.5 (3)	N5—C26—N6—C27	−6.0 (4)
C19—C20—C21—C22	−178.9 (2)	S2—C26—N6—C27	175.02 (18)
C20—C21—C22—C23	0.3 (4)	C28—C27—N6—C26	160.2 (2)
C21—C22—C23—C24	−0.2 (4)	C32—C27—N6—C26	−24.6 (4)
C22—C23—C24—C25	−0.8 (4)	N2—C10—S1—Pd1	−6.03 (18)
C23—C24—C25—C20	1.7 (4)	N3—C10—S1—Pd1	174.54 (14)
C21—C20—C25—C24	−1.5 (4)	N5—C26—S2—Pd1	1.1 (2)
C19—C20—C25—C24	178.0 (2)	N6—C26—S2—Pd1	179.99 (15)

### Hydrogen-bond geometry (Å, º)

**Table:**

*D*—H···*A*	*D*—H	H···*A*	*D*···*A*	*D*—H···*A*
C1—H1···S2	0.93	2.60	3.230 (2)	126
C16—H13···N2	0.93	2.32	2.887 (3)	119
C17—H14···S1	0.93	2.72	3.355 (2)	126
C32—H26···N5	0.93	2.39	2.911 (3)	115
N3—H27···S2^i^	0.86	2.63	3.4805 (18)	171
N6—H28···S1^ii^	0.86	2.84	3.6554 (19)	159

Symmetry codes: (i) *x*, −*y*+1/2, *z*+1/2; (ii) *x*, −*y*+1/2, *z*−1/2.
